# Serum Uric Acid and Subsequent Cognitive Performance in Patients with Pre-Existing Cardiovascular Disease

**DOI:** 10.1371/journal.pone.0120862

**Published:** 2015-03-20

**Authors:** Noa Molshatzki, Galit Weinstein, Jonathan Y. Streifler, Uri Goldbourt, David Tanne

**Affiliations:** 1 Sagol Neuroscience Center, Chaim Sheba Medical Center, Tel Hashomer, Israel; 2 Department of Neurology, Boston University School of Medicine, Boston, Massachusetts, United States of America; 3 Neurology Unit, Rabin Medical Center, Golda campus, Petach Tikva, Israel; 4 Department of Epidemiology and Preventive Medicine, School of Public Health, Sackler Faculty of Medicine, Tel Aviv University, Tel-Aviv, Israel; Texas Tech University Health Science Centers, UNITED STATES

## Abstract

High serum uric acid (UA) levels are associated with numerous vascular risk factors, and vascular disease, that predispose patients to cognitive impairment, yet UA is also a major natural antioxidant and higher levels have been linked to slower progression of several neurodegenerative disease. In-order to test the association between UA and subsequent cognitive performance among patients that carry a high vascular burden, UA levels were determined by calorimetric enzymatic tests in a sub-cohort of patients with chronic cardiovascular disease who previously participating in a secondary prevention trial. After an average of 9.8±1.7 years, we assessed cognitive performance (Neurotrax Computerized Cognitive Battery) as well as cerebrovascular reactivity (CVR) and common carotid intima-media thickness (IMT). Among 446 men (mean age 62.3±6.4 yrs) mean UA levels were 5.8±1.1 mg/dL. Adjusted linear regression models revealed that low UA levels (bottom quintile) were associated with poorer cognitive performance. Adjusted differences between the bottom quintile and grouped top UA quintiles were (B coefficient±SE) −4.23±1.28 for global cognitive scores (p = 0.001), −4.69±1.81 for memory scores (p = 0.010), −3.32±1.43 for executive scores (p = 0.020) and −3.43±1.97 for visual spatial scores (p = 0.082). Significant difference was also found for attention scores (p = 0.015). Additional adjustment for impaired CVR and high common carotid IMT slightly attenuated the relationship. Stronger UA effect on cognitive performance was found for older (age>65) patients with significant age interaction for global cognitive score (p = 0.016) and for executive (p = 0.018) and attention domains (p<0.001). In conclusion, we demonstrate that low UA levels in patients with preexisting cardiovascular disease are associated with poorer cognitive function a decade later. These findings lend support to the hypothesis that oxidative stress may be involved in the pathogenesis of age-associated cognitive impairment.

## Introduction

There are a plethora of epidemiological studies linking high serum uric acid (UA) levels with increased risk of vascular disease [1–3]. High UA levels are also associated with major vascular risk factors such as hypertension [4], chronic kidney disease [5], the metabolic syndrome and diabetes [6], as well as with cerebral ischemic white matter changes [7] and carotid atherosclerosis [8] that all predispose to vascular cognitive impairment.

Because of its higher levels in humans in comparison to other mammals (due to uricase inactivation) and radical scavenging properties, UA is also a major natural antioxidant and higher levels have been linked to slower progression of several neurodegenerative disease [9, 10]. Therefore, the different properties of UA might have conflicting effects on the risk of cognitive function in which both vascular mechanisms and oxidative stress are thought to play a role. In the Rotterdam Study, an association was found between higher UA levels and better global cognitive, executive and memory functions later in life. While age and gender adjusted analyses showed no clear association, adjustment for cardiovascular risk revealed the possible protective effect of higher UA levels [11]. On the other hand, in a cross-sectional analysis conducted on a subsample of the Rotterdam study participants, high UA levels were associated with white matter atrophy and with poorer cognition [12].

Patients with cardiovascular disease (CVD) carry a high vascular burden and are prone to a high risk of vascular cognitive impairment [13]. We hypothesized, therefore, that among patients with pre-existing CVD, higher UA levels would be associated with poorer cognitive function. We have assessed cognitive functions and ultrasound indices of cerebrovascular disease in a sub-cohort of patients who had previously participated in a clinical trial of lipid modification (Bezafibrate Infarction Prevention; BIP) [14]. This provided the opportunity to examine among patient with long-lasting CVD the association between levels of serum UA and specific domains of cognitive performance assessed a decade later.

## Methods

### Study Population

The study sample included surviving participants from 7 central hospitals who reside in the central region of Israel and who previously participated in the BIP study. The BIP study was a placebo-controlled randomized clinical trial conducted during 1990–1997 that has investigated the efficacy of bezafibrate retard 400 mg daily, a fibric derivative, in secondary prevention among patients with established stable CVD [14]. In brief, patients were recruited in 18 medical centers and included predominantly men 45 to 74 years of age with myocardial infarction at least 6 months and not longer than 5 years before enrollment, and/or stable angina pectoris during the last 2 years confirmed by coronary angiography, and/or radionuclide studies or standard exercise tests. A lipid profile of serum total cholesterol between 180 and 250 mg/dL, low-density lipoprotein cholesterol≤180 mg/dL (≤160 mg/dL for patients <50 years), high-density lipoprotein cholesterol≤45 mg/dL and triglycerides ≤300 mg/dL was required. Main exclusion non-lipid criteria were insulin-dependent diabetes mellitus, hepatic or renal failure, and disabling stroke. Further details of the study have been described elsewhere [14].

**Fig 1:**
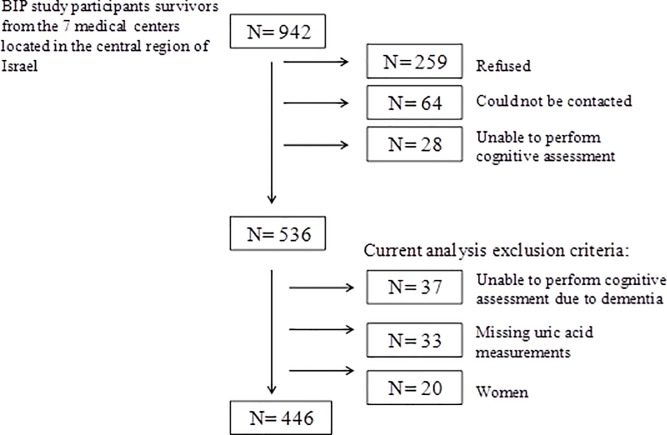
Flow diagram of study participants.

Fig. 1 describes the study population. Between 2004 and 2008, surviving patients were invited for a late-life evaluation included medical and cognitive function assessments (n = 942). The evaluation took place at a central research center (Sagol Neuroscience Center), or if unable or unwilling to attend the medical center, patients were assessed at their residence. Of patients invited, 259 refused to participate, 64 could not be contacted and 28 were unable to perform the cognitive assessment. We further excluded 55 individuals with incomplete cognitive data, 37 who were unable to perform cognitive assessment due to dementia, 35 subjects with missing UA measurements and 20 women (since represented <5% of patients and women tend to have lower UA levels).

### UA and Baseline Measurements

Blood samples were collected after ≥12 hours of fasting using standardized equipment and procedures. Serum analysis was carried out in a central laboratory using standard automated procedures with commercially available diagnostic kits (Boehringer Mannheim, Mannheim, Germany). Accuracy and precision were under periodic surveillance by the Centers for Disease Control/National Heart, Lung, and Blood Institute’s lipids standardization program (Bethesda, Maryland) and by the Wellcome-Murex diagnostic clinical chemistry quality assessment program. UA levels used were determined by calorimetric enzymatic tests assessed at the final evaluation of the BIP trial [15].

Education level was obtained at enrollment to the BIP trial and all biochemical and clinical measurements used in the current analysis were from last BIP trial evaluation. Chronic kidney disease was defined following the National Kidney Foundation definition as kidney damage, reflected by an estimated glomerular filtration rate of less than 60 ml/min/1.73 m^2^ of body-surface area, estimated using the 4-variable Modification of Diet in Renal Disease formula [16].

### Cognitive Assessment

Cognitive functions were assessed using a validated set of computerized cognitive tests (Neurotrax Computerized Cognitive Battery) as part of the late-life evaluation [17].

The cognitive assessment examines a wide range of cognitive domains, including memory (verbal and non-verbal), executive function, visual spatial skills and attention. A global score was computed, summarizing performance of four cognitive domains. The tests are designed for the elderly and they are interactive and adaptive. Guidance and feedback was provided in the practice sessions that precede each test, but not during the actual tests. Cognitive scores were normalized according to age and education specific normative data. Normalized scores were then scaled to an IQ-style scale with mean of 100 and SD of 15. Out of 446 patients 440 had memory records, 432 had executive function records, 434 had visual spatial skills records, 439 had attention records and 434 had global score records.

### Indices of Cerebrovascular Disease

As part of late-life evaluation patients evaluated at the central research center underwent ultrasound cerebrovascular evaluations assessing cerebral large vessel atherosclerosis and small vessel occlusive disease: i. Ultrasound examination for common carotid intima-media thickness (IMT) measurement. IMT was measured at the far wall of both common carotid arteries using high resolution B-mode ultrasound. Assessment followed a standard protocol that includes acquiring images of both carotid arteries and measurement thickness employing automatic edge detection (METRIS, France). High IMT was defined as the top quartile; >1.06 mm. In addition, the presence of plaque was recorded on both sides. ii. Cerebrovascular reactivity (CVR), a marker of cerebral microvascular dysfunction, was evaluated by Transcranial Doppler (TCD) (Rimed Trans-link 9900 TCD, Herzliya, Israel) using the breath-holding index [18]. BHI was calculated as: [mean flow velocity at the end of breath holding minus mean flow velocity at rest divided by mean flow velocity at rest] multiplied by 100 divided by seconds of breath holding. Impaired CVR was defined as the lower quartile of BHI; BHI<0.47.

### Data Analysis

UA levels, assessed at the final evaluation of the BIP trial, were divided into quintiles. Univariate associations between UA quintiles and other demographic and clinical variables were evaluated with analysis of variance (ANOVA), Chi Square or Kruskal Wallis tests. Unadjusted means and 95% CI of cognitive scores were calculated across UA quintiles. Linear regression models were used to identify potential association between UA and cognitive scores adjusted for age, educational level, high-density lipoprotein cholesterol, chronic kidney disease, diabetes and BIP study arm (Model 1) and in addition to impaired CVR and high CC-IMT (Model 2). Patients at the bottom quintile of UA (<4.8 mg/dL) demonstrated poorer cognitive function with p<0.05 for the memory, visual spatial domains and for global score. The top four UA quintiles were grouped together due to comparable cognitive scores outcomes and were compared to the bottom quintile.

Analysis was repeated separately for several subgroups divided by key risk factors: presence of diabetes, CKD, and age groups (with 65 years as the cutting point). Transformation of power of 4 was used for the attention cognitive domain to approximate normality. Hence, for attention cognitive domain unadjusted means are not presented and only p values are tabulated for linear regression models. Multiple imputation [19] was used to generate imputed values for impaired CVR and high common carotid IMT (total of 70 records). Analyses were performed with R statistical software version 2.13.2 [20].

### Ethics statement

The study was approved by the Ethics Committee of the Chaim Sheba Medical Center. Written informed consent was obtained from all participants. Data handling and analysis were performed by study identification codes to preserve anonymity.

## Results

Among the 446 male patients mean age at last BIP trial evaluation was 62.3±6.4 years and mean UA levels were 5.8±1.1 (range from 2.3 to 10.0) mg/dL. Compared with participants who were excluded from the analyses, those who were included had a higher proportion of men to women and were younger and more educated. Baseline characteristics of the study cohort across UA quintiles are shown in Table 1. Decreasing quintiles of UA were associated with lower education, more diabetes, and less CKD and there was a borderline association with high-density lipoprotein cholesterol.

**Table 1:** Study characteristics according to uric acid quintiles.

	Uric Acid Levels Quintiles (mg/dL)					
	< 4.84 n = 90	4.84–5.43 n = 90	5.44–6.01 n = 88	6.02–6.76 n = 90	>6.76 n = 88	p for trend
**At baseline**						
Age	61.9+−6.5	62.9+−7.2	63.2+−5.9	61.5+−6.3	62.2+−6.3	0.71
Educational level						
Primary	26 (27.7)	26 (26.8)	14 (15.4)	12 (12.8)	17 (18.3)	0.007
Secondary school	44 (46.8)	49 (50.5)	47 (51.6)	52 (55.3)	43 (46.2)	
Intermediary or University	24 (25.5)	22 (22.7)	30 (33.0)	30 (31.9)	33 (35.5)	
Systolic BP (mm Hg)	129.3+−15.6	131.5+−18.3	131.3+−17.4	132.9+−21.4	129+−16.1	0.89
Diastolic BP (mm Hg)	78.2+−8.1	77.6+−8	78.1+−8.8	79.8+−9.8	78.7+−8.1	0.26
Body mass index (kg/m^2^)	26.1 (24.6–28.5)	27.1 (24.8–28.7)	26.4 (24.4–28.4)	26.8 (25–29.7)	27.3 (25.3–29.4)	0.21
Total cholesterol (mg/dL)	214.4+−24.7	205.6+−28.5	207.9+−29.4	203.8+−28.8	210.8+−26.5	0.33
LDL cholesterol (mg/dL)	149.1+−21.8	142.1+−25.9	143.3+−25.1	138.3+−24.7	145.9+−25.2	0.21
HDL cholesterol (mg/dL)	41+−9.3	38.2+−7.6	39.8+−7.6	39+−8.2	38.4+−7.6	0.09
Triglycerides (mg/dL)	109.8 (78–150.2)	116.2 (87.6–159.7)	110.7 (84.7–150.5)	119.5 (90.1–157.1)	123.7 (88.9–166.3)	0.40
Glucose (mg/dL)	95.3 (86.6–113)	90.7 (84.8–97.9)	90.7 (84.7–109.3)	91.4 (83.9–104.2)	94.3 (87.2–103.4)	0.084
Fibrinogen (mg/dL)	311.5 (274.8–343.1)	303.1 (270.4–341.3)	301.7 (268.5–341.6)	289.5 (264.3–324)	293.1 (257.8–327.7)	0.23
White blood count (10^9/L)	6.4 (5.4–7.5)	6.4 (5.3–7.3)	6.7 (5.5–7.6)	6.4 (5.5–7)	6.6 (5.7–7.8)	0.31
Past myocardial infraction	65 (72.2)	75 (83.3)	69 (78.4)	72 (80.9)	72 (81.8)	0.22
Diabetes	28 (31.1)	10 (11.1)	17 (19.3)	13 (14.4)	13 (14.8)	0.022
Past stroke	2 (2.2)	2 (2.2)	2 (2.3)	4 (4.4)	4 (4.5)	0.24
Chronic kidney disease	9 (10)	24 (26.7)	19 (21.6)	25 (27.8)	41 (46.6)	<0.001
**Late life evaluation**						
Impaired CVR	29 (34.5)	19 (23.8)	16 (21.9)	13 (16)	15 (19)	0.008
High CC-IMT	30 (35.7)	19 (22.9)	16 (20.3)	25 (30.5)	13 (15.5)	0.026
Bilateral carotid plaque	45 (52.3)	51 (60)	48 (60)	43 (51.8)	42 (50)	0.47

Values are N (%) for categorical variables, mean ±SD for continuous variables and median (IQR) for continuous skewed variables.

BP, blood pressure; CC-IMT, common carotid intima-media thickness; CVR, cerebrovascular reactivity; HDL, high-density lipoprotein; LDL, low-density lipoprotein.

**Fig 2:**
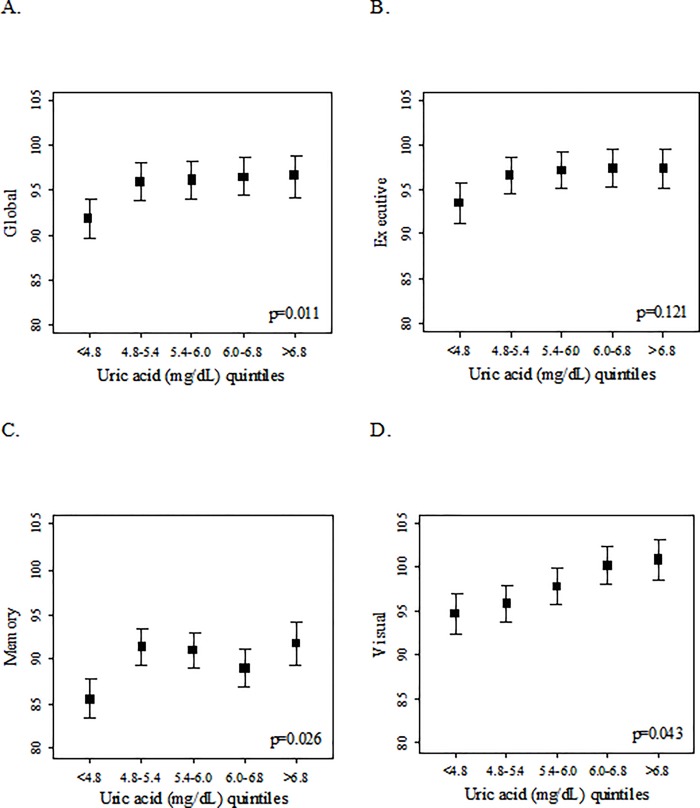
Unadjusted means and 95% CI of cognitive global (panel A), executive function (panel B), memory (panel C) and visual (panel D) scores by uric acid quintiles. Since transformation of power of 4 was required for the attention cognitive domain to approximate normality a figure of unadjusted means for the attention domain is not presented. P value for the attention cognitive domain is 0.15.

Late-life evaluation was conducted after an average of 9.8 ±1.7 years after the last BIP trial evaluation. Decreasing quintiles of UA were associated with higher rates of impaired CVR and high common carotid IMT. Unadjusted means and 95% CI of cognitive scores by UA quintiles are depicted in Fig. 2.

Adjusted linear regression models (Table 2) demonstrated that low UA levels were associated with poorer cognitive performance a decade later. Adjusted differences between bottom vs. higher UA quintiles were (B coefficient ± SE) −4.23±1.28 for global cognitive scores (p = 0.001), −4.69±1.81 for memory scores (p = 0.010), −3.32±1.43 for executive scores (p = 0.020) and −3.43±1.97 for visual spatial scores (p = 0.082). Significant difference was also found for attention scores (p = 0.015). Additional adjustment for impaired CVR and high common carotid IMT slightly attenuated the relationship. Similar results were obtained in a sensitivity analysis excluding subjects on diuretics (n = 26, data not shown), since diuretic may induce hyperuricemia.

**Table 2:** Linear Regression Model of association between low UA (bottom quintile vs. higher) and cognitive performance.

Cognitive domain	Model 1		Model 2	
	B coefficient ±SE	p	B coefficient ±SE	p
Global	−4.23 ± 1.28	0.001	−3.77 ± 1.29	0.004
Memory	−4.69 ± 1.81	0.010	−4.38 ± 1.82	0.017
Executive	−3.32 ± 1.43	0.020	−2.62 ± 1.43	0.070
Visual Spatial	−3.43 ± 1.97	0.082	−3.31 ± 2.00	0.100
Attention	*	0.015	*	0.057

Model 1: Adjusted for age, educational level, HDL cholesterol, chronic kidney disease, diabetes and BIP study arm.

Model 2: Adjusted in addition to impaired CVR and high CC-IMT.

* Transformation of power of 4 was used for attention cognitive domains to approximate normality. Hence, only p values are tabulated.

Linear regression models divided by key risk factors for cognitive impairment are presented in Table 3. Stronger UA effect on cognitive performance was found for older (age>65) patients with significant age interaction for global cognitive score (p = 0.016) and for executive (p = 0.018) and attention domains (p<0.001).

**Table 3:** Linear Regression Model of association between low UA (bottom quintile vs. higher) and cognitive performance for various subgroups.

	B coefficient ±SE	p	B coefficient ±SE	p	Int.
Age	Age >65 (N = 173)		Age ≤65 (N = 298)		
Global	−8.86 ± 2.42	<0.001	−2.23 ± 1.50	0.14	0.016
Memory	−8.35 ± 2.70	0.003	−3.02 ± 2.35	0.20	0.14
Executive	−8.85 ± 2.86	0.002	−1.16 ± 1.62	0.48	0.018
Visual Spatial	−0.81 ± 3.71	0.83	−4.50 ± 2.35	0.056	0.35
Attention	*	<0.001	*	0.89	<0.001
Chronic kidney disease	Yes (N = 128)		No (N = 343)		
Global	−6.84 ± 3.80	0.075	−3.64 ± 1.36	0.008	0.26
Memory	−7.74 ± 5.01	0.13	−4.16 ± 1.97	0.035	0.37
Executive	−7.12 ± 4.31	0.102	−2.64 ± 1.51	0.082	0.22
Visual Spatial	−2.95 ± 5.92	0.62	−3.31 ± 2.11	0.12	0.87
Attention	*	0.24	*	0.042	0.38
Type 2 diabetes	Yes (N = 88)		No (N = 383)		
Global	−1.90 ± 2.48	0.45	−4.79 ± 1.48	0.001	0.52
Memory	−3.43 ± 3.41	0.32	−4.98 ± 2.13	0.020	0.93
Executive	−2.68 ± 2.82	0.35	−3.21 ± 1.65	0.053	0.78
Visual Spatial	−0.06 ± 3.88	0.99	−4.40 ± 2.29	0.066	0.45
Attention	*	0.71	*	0.009	0.29

All models adjusted to age, educational level, HDL cholesterol, chronic kidney disease, type 2 diabetes and BIP study arm.

* Transformation of power of 4 was used for the attention cognitive domain to approximate normality. Hence, only p values are tabulated.

## Discussion

Our main finding is that among men with long-lasting CVD and a high vascular burden, contrary to our hypothesis, low UA levels are associated with poorer cognitive functions assessed a decade later. Associations were found for memory, executive and attention domains and for a global cognitive score. An interaction with age was identified such that in older persons a stronger association of low UA with poorer cognitive performance was observed.

Serum UA may have conflicting associations with late-life cognitive functioning. A substantial body of evidence supports the concept of cognitive impairment as a vascular disorder [21, 22]. Oxidative stress and the consequent accumulation of free radicals play also an important role in the neuropathologic manifestations of cognitive impairment [23, 24]. Higher UA levels have been linked to slower progression of several neurodegenerative diseases [9, 10, 25, 26]. Apparently healthy subjects with higher UA levels included in the Rotterdam study exhibited better global cognitive, executive and memory functions later in life [11]. High UA levels were also found to be associated with a reduced rate of cognitive decline [27, 28] yet other studies have found an opposite trend [29, 30].

The inconsistency of the association between UA and cognitive function may be explained in-part by the time in which the UA levels were measured. Keeping in mind that cognitive impairment is a disease with a long induction period, a long period between the UA measurement and the cognitive evaluation is required in order to assess a relationship. It is possible that measuring UA levels years before cognitive deterioration occurs may be more accurate than measuring it cross-sectionally, as is the case with other risk factors related to cognition such as hypertension, diabetes and obesity.

The brain is unique with regard to its great vulnerability to oxidative damage due to its high metabolic rate. Neurons are therefore exposed to an environment with considerable production of reactive oxygen species compared to other cellular systems of other organs. The antioxidant proprieties of UA have been extensively characterized in vitro where it was found to be a peroxynitrite scavenger and to form stable complex with iron ions, reducing their oxidant potential [31, 32]. Further, a neuroprotective effect and a facilitative role for astrocytes in the neuroprotective effect of urate have been suggested [33]. Genetic evidence on the association between UA transporter gene (SLC2A9) and memory performance found in the Lothian Birth Cohort shed further support for the possible association between higher UA levels and better cognitive function later in life [34].

Thus we are facing a paradox, in which, on one hand, UA is good for the preservation of brain functions and on the other hand high UA is associated with an increased risk of vascular brain disease including strokes. This problem is hard to disintegrate as the two conditions often coexist. Our study is unique in testing cognitive performance among patients with long-lasting CVD and contrary to our hypothesis, low rather than high UA levels were associated with poorer cognitive functions.

Strengths of our study include the focus on a high-risk population of individuals with long-lasting CVD, the use of a validated computerized assessment tool to quantify cognitive function in specific domains, and evaluation for ultrasound indices of cerebral small-vessel and large-vessel disease. Limitations include a single cognitive assessment, lack of systematic brain imaging and the fact that analyses are restricted to men. We also lack follow-up measurements of UA levels, yet serum UA levels are highly heritable and repeated measures in the same individual are fairly stable (intraclass correlation coefficient (ICC) 0.8 [27]. Although UA appears to have the potential for neuroprotection, it is possible that the predictive association between low UA and cognitive impairment reflects instead the effect of a urate precursor, such as adenosine or inosine, or another determinant of systemic urate concentrations. Finally, our analyses could be prone to an index event bias, since all subjects were included based on the occurrence of established stable CVD [35]. Our findings are, however, in line with findings from a population based study and with a large body of evidence regarding the neuroprotective effects of UA.

To summarize, despite mounting evidence regarding the association between high UA and vascular disease, we have observed among patients with long-lasting CVD an association between low UA (bottom quintile) and cognitive impairment a decade later. A stronger association between low UA and poorer cognitive performance was found for older patients lending support to the hypothesis of an age-associated increase in oxidative damage involved in the pathogenesis of cognitive impairment.

Implications: Serum UA levels are linked to hypertension, renal disease, and CVD. Several strategies to lower UA as a novel method of reducing the burden of cardiovascular and cerebrovascular disease have been proposed. The current findings have important implications for the way we view high UA and for future therapeutic interventions.
